# Fever Dreams: An Online Study

**DOI:** 10.3389/fpsyg.2020.00053

**Published:** 2020-01-28

**Authors:** Michael Schredl, Daniel Erlacher

**Affiliations:** ^1^Sleep Laboratory, Central Institute of Mental Health, Medical Faculty Mannheim, Heidelberg University, Mannheim, Germany; ^2^Institute of Sport Science, University of Bern, Bern, Switzerland

**Keywords:** dreaming, fever, heat perception, continuity hypothesis, fever dreams

## Abstract

In addition to a large variety of somatic symptoms, fever also affects cognition, sleep, and mood. In an online survey with 164 participants, 100 fever dream reports were submitted. Fever dreams were more bizarre and more negatively toned and included more references to health and temperature perception compared to “normal” most recent dreams – findings that are in line with the continuity hypothesis of dreaming. Future studies should follow up this line of research by conducting diary studies during naturally occurring febrile illnesses and sleep laboratory studies with experimentally induced fever. It would also be very interesting to study the effect of thermal stimulation applied during sleep on dream content. This research helps to understand subjective experiences while sleeping in an extreme condition (elevated body temperature).

## Introduction

Fever is an elevation of body temperature that exceeds the normal daily variation and is based on an increased hypothalamic set point (Dinarello and Porat, 2015). Typical symptoms are runny or dripping nose, sore throat, trouble with breathing, weakness, feeling hot and/or feeling cold, sweating, and chills (Ames et al., 2013). In addition to symptoms such as headache, malaise, lack of appetite, and other sickness-related disorders (Ogoina, 2011) sleep might be disturbed (Powers et al., 2015). Drake et al. (2000) found reduced sleep efficiency in those participants who developed moderate symptoms of a common cold (7 out of 21 participants) in response to an experimentally introduced rhinovirus. An actigraphy study (Smith, 2012b), however, monitoring 15 participants suffering from a common cold, found only small or no significant sleep disturbances; only those persons who reported nasal obstruction as a major symptom had reduced sleep efficiency. Higher temperatures of about 39°C during sleep (experimentally induced via pyrogens) significantly increased wake time and reduced slow wave and rapid eye movement (REM) sleep (Karacan et al., 1968). In a single subject with very high fever (40.5°C at the beginning of the night to 39.2°C in the morning) frequent awakenings and no REM sleep were recorded during the 7 h of sleep (Maron et al., 1964). REM sleep reducing effects were also reported using experimentally applied endotoxin to stimulate the immune system (Pollmacher et al., 1993; Mullington et al., 2000). In addition to the somatic symptoms associated with fever, negative moods and cognitive impairments like psychomotor slowing and lower working memory performance can accompany common colds with fever (Hall and Smith, 1996; Smith, 2012a). Even small increases in body temperature induced by experimentally administered endotoxin can impair cognitive performance and increase depressive mood (Reichenberg et al., 2001).

Based on the effects of fever on sleep and cognition, one might expect that fever also affects dreaming defined as subjective experience during sleep. Karacan et al. (1968) reported that dream recall after fever nights (free recall in the morning) was much lower (17%) than recall after baseline and recovery nights (about 80%) which fits in with the findings of reduced REM sleep (Pollmacher et al., 1993; Mullington et al., 2000) as awakenings from REM sleep are related to higher dream recall (Nielsen, 2000) and reduced working memory present in fever (Smith, 2012a) might affect the ability to recall a dream. Regarding the content of dreams associated with fever, Ames et al. (2013) found that 11% of the 28 participants reported unusual, strange dreams accompanying their fever, e.g., “back and forth between a very difficult circumstance and a very comfortable circumstance.” Analyzing 46 retrospectively recalled fever dreams, Schredl et al. (2016b) found that fever dreams are more bizarre and more negatively toned compared to everyday dreams. This is in line with the continuity hypothesis of dreaming (Domhoff, 2003; Schredl, 2003) which states that dreams reflect all kind of waking-life experiences, like concerns, thoughts, actions, etc. since the negative dream emotions are related to the negative daytime mood and the bizarreness to the cognitive impairments, e.g., working memory, related to fever. Common themes in fever dreams were spatial distortions, e.g., moving walls, creatures with over-sized arms and legs, and threats (dogs, big spheres, insects, terrorists) (Schredl et al., 2016b). More detailed content analytic studies on fever dreams, however, have not been carried out so far.

The aim of the present study was to extend the findings of the previous pilot study (Schredl et al., 2016b) where we only looked at dream bizarreness and global dream topics in fever dreams and to carry out a more sophisticated dream content analysis including dream emotions, dream characters, interactions, aggression, and especially health-related themes and explicit temperature perception within the dream in a completely new data set of fever dreams. According to the continuity hypothesis, we expected more bizarre dreams, more negative and less positive dream emotions, and more dreams including references to perception of heat. As fever is present during sleep, increase in heat perceptions in the dream might reflect incorporation of the internal stimuli of elevated body temperature (cf. Nielsen, 2017).

## Materials and Methods

### Participants

The sample included 164 participants (63 women and 101 men) with a mean age of 22.90 ± 9.02 years, ranging from 12 to 56 years. The participation was voluntary and without monetary compensation. Ninety persons reporting a most recent fever dream (39 women and 51 women; age mean: 23.57 ± 8.03 years) were matched with 90 persons reporting a most recent dream in the study of Schredl et al. (2010–2011) according to age, gender, and dream length. That is, the gender distribution was exactly the same and the age mean was 23.67 ± 8.15 years (due to matching ± 1 year).

### Questionnaire

Besides demographic data, a seven-point scale (coded as 0 = never, 1 = less than once a month, 2 = about once a month, 3 = about two to three times a month, 4 = about once a week, 5 = several times a week, 6 = almost every morning) for measuring dream recall frequency was presented (Schredl, 2004); the retest reliability is high *r* = 0.85 (mean interval about 8 weeks). The overall emotional intensity of the remembered dreams in general was measured on a five-point scale (0 = not at all intense, 1 = not that intense, 2 = somewhat intense, 3 = quite intense, 4 = very intense). The retest reliability of this scale is fairly high (*r* = 0.704; Schredl et al., 2014).

The frequency of having fever was elicited using a five-point scale: 0 = never, 1 = once, 2 = twice or three times, 3 = about once a year, and 4 = more than once a year. One question aimed at the frequency of fever dreams if fever is present: 0 = never, 1 = less than half the days with fever, 2 = about half the days with fever, 3 = more than half the days with fever, and 4 = every day with fever.

Then the participants were asked to write down the last fever dream they remembered as completely as possible. The instructions for the matched sample reporting a “normal” most recent dream were similar (Schredl et al., 2010–2011). Furthermore, questions were presented about how long ago the fever dream occurred (0 = last week, 1 = last month, 2 = 1–2 months ago, 3 = 3–6 months ago, 4 = 6 months to 1 year ago, 5 = 1–2 years ago, 6 ≥ 2 years ago) and the emotional tone of the fever dream (predominantly positive, neutral/balanced, predominantly negative). Lastly, the overall emotional intensity of all remembered fever dreams were to be rated on a five-point rating scale (see rating scale of overall emotional intensity of all remembered dreams).

### Dream Content Analysis

The dream content analytic scales were adopted from Schredl et al. (1998a, c): Bizarreness/realism (1 = possible in waking life and dream events are part of normal everyday life, 2 = many elements of waking life, but the dream action is uncommon but not impossible, 3 = occurrence of one fantasy object, a bizarre connection, or action which is impossible in waking life, and 4 = occurrence of two or more fantasy objects, bizarre connections, or actions which are impossible in waking life), positive and negative emotions (two four-point scales: 0 = none, 1 = mild, 2 = moderate, 3 = strong), number of dream characters, verbal interaction (Yes/No), physical interaction like fighting, caressing (Yes/No), occurrence of verbal or physical aggression (Yes/No), and occurrence of health-related dreams. The interrater reliabilities of the scale were ranging from *r* = 0.689 to 0.779 (Schredl et al., 2004). For the nominal scales the exact agreement indices ranged from 76.3 to 95.9% (Schredl et al., 1998c). For the purpose of this study, a new scale was constructed, i.e., whether or not the dreamer experienced some kind temperature perception within the dream.

### Procedure

Ethics approval and parental consent were not required for this student project (carried out online posted at a freely accessible website, i.e., not aiming at including adolescents, and not including distressing questions) as per the University of Mannheim’s guidelines and applicable institutional and national guidelines. The informed consent of the participants was implied through survey participation and completion. The questionnaire was posted on “klartraumforum.de,” a website addressing lucid dreamers and person with interest in lucid dreams. The dreams were checked and all elements not related to the dream experience were removed. Ninety most recent dreams from the study by Schredl et al. (2010–2011) were selected, matched for word count, gender, and age. Ten fever dreams could not be matched. The 190 dreams were sorted in a random order to ensure that the raters did not know whether the dream was a fever dream or a control dream. One rater applied the dream content scales (see the section “Dream Content Analysis”). Statistical analysis was executed using SAS 9.4 for Windows. According to the scales’ measurement levels (interval, ordinal, or nominal), we computed *t*-tests, Mann–Whitney *U*-tests, and Chi-square test. In addition, a logistic regression (cumulative logit analyses) was performed to identify factors associated with reporting a most recent fever dream.

## Results

Mean dream recall frequency was 4.67 ± 1.49; corresponding to the category “several times a week.” The mean emotional intensity of all remembered dreams was 2.80 ± 0.90.

Ten participants reported that they never had fever, 10 participants reported having fever once, 31 twice or three times, 91 about once a year, and 20 more than once a year (two missing values). The frequency of fever dreams while having fever is depicted in Table 1. One-fifth of the participants never experienced dreams while having fever, but most participants did, even quite frequently. The mean emotional intensity of fever dreams was 2.92 ± 1.43 (*N* = 150). They were significantly more intense than dreams in general (difference: 0.13 ± 1.67, *N* = 149; sign rank test: *N* = 17, *p* = 0.0022).

**TABLE 1:** Percentage of fever dreams while having fever (*N* = 152).

**Variable**	***N***	**Percentage**
Every day with fever	24	15.79
More than have the days with fever	30	19.74
About half the days with fever	29	19.08
Less than half the days with fever	37	24.34
Never	32	21.05

Most recent fever dreams were reported by 100 participants (41 women, 59 men) with the mean age of 22.64 ± 8.12 years. Mean word count was 65.24 ± 77.34 words (5–482 words). The time intervals between the most recent fever dream and reporting the dream for the study were distributed as follows: last week (*N* = 7), last month (*N* = 5), 1–2 months ago (*N* = 9), 3–6 months ago (*N* = 11), 6 months to 1 year ago (*N* = 17), 1–2 years ago (*N* = 17), and >2 years ago (*N* = 34). Reporting a most recent fever dream did not depend on dream recall frequency but on fever frequency (Table 2). Moreover, the time interval between the most recent fever dream and reporting the fever dream was not related to realism/bizarreness, positive and negative dream emotions, and the other variables; solely, the correlation (Spearman Rank correlation) between the time interval and temperature perception dreams was negative. The more recent dreams included temperature perceptions more often than dreams experienced a long time ago.

**TABLE 2:** Factors affecting the report of a most recent fever dreams (Yes/No).

**Variable**	**SE**	**Wald *X*^2^**	***p***
Age	−0.0178	0.0	0.8522
Gender	0.1192	1.2	0.2662
Dream recall frequency	0.0386	0.1	0.7069
Fever frequency	0.3503	11.9	0.0006

*SE, standardized estimate (logistic regression).*

The comparison between fever dreams and most recent dreams is depicted in Table 3. Since one of the matching criteria was dream length, the mean word counts between the two samples were almost identical. In accordance with the hypotheses (see the section “Introduction”), fever dreams were more bizarre, included fewer positive emotions but more negative emotions than most recent dreams. The number of dream persons and interactions (verbal and physical) is lower in fever dreams, whereas no difference occurred for aggression as a dream topic. Fever dreams included more health-related topics and more often the perception of temperature in dreams (see dream examples). Using the Holm–Bonferroni correction – as we tested four hypotheses (bizarreness, positive and negative emotions, temperature perception) – the findings remain significant.

**TABLE 3:** Fever dreams and control dreams.

**Variable**	**Fever dreams (*N* = 90)**	**Control dreams (*N* = 90)**	**Stat. tests^1^**
Word count	66.74 ± 79.61	67.94 ± 81.64	*t* = −0.1, *p* = 0.9206
Bizarreness/realism	2.78 ± 0.94	2.13 ± 0.74	*z* = 5.9, *p* < 0.0001^2^
Positive emotions	0.39 ± 0.93	0.96 ± 1.20	*z* = −3.6, *p* < 0.0001^2^
Negative emotions	2.22 ± 1.00	1.39 ± 1.22	*z* = 4.5, *p* < 0.0001^2^
Number of persons	0.51 ± 0.89	1.44 ± 1.36	*t* = −5.5, *p* < 0.0001
Verbal interaction (Yes/No)	11.11%	28.89%	*X*^2^ = 8.9, *p* = 0.0029
Physical interaction (Yes/No)	4.44%	18.89%	*X*^2^ = 9.1, *p* = 0.0025
Aggression (Yes/No)	18.89%	15.56%	*X*^2^ = 0.4, *p* = 0.5537
Health-related topic (Yes/No)	11.11%	3.33%	*X*^2^ = 4.1, *p* = 0.0438
Temperature perception (Yes/No)	10.00%	2.22%	*X*^2^ = 4.7, *p* = 0.0147^2^

*^1^Statistical tests included *t*-tests, Mann–Whitney *U*-test, and Chi-square test with the test statistics *t*, *z*, and **X**^2^ and the probability *p* (significant findings with *p* < 0.05). ^2^One-tailed.*

### Fever Dream Examples Including Heat Perception

“I was in my room sleeping and awakened because my body felt as if it was blazing. I tried to uncover my body and to drink something but I felt too weak to do so. My mother came in but she could not help either. I tried to move and pull off the bedspread but it didn’t work. The most intense feelings were weakness and helplessness.”

“I am walking in a city that is situated in a valley, maybe Italy. It is dusk and a somewhat cool breeze is present. I have a shawl around my shoulders which I pulled around me. I planned a relaxing walk before bedtime. Suddenly, a hot wind has sprung up. I do not know where I am (in the city) and it is growing steadily hotter. I lost my shawl, and also my shoes; I feel the relatively cool cobble stones of the street. I have the impression that I have to run away quickly. The air is so hot that breathing hurts. A gut feeling tells me it is not good to stay outside, so I start running, not knowing where I am going. Everything around me is unfamiliar, the houses become enormous, and a muffled thunder is coming from the nearby mountain. I see a red glow in the corner of my eyes and I turn around to see an enormous lava ball coming down the mountain heading toward the city and me. I am running faster and faster, the air grows hotter and hotter, the lava ball is changing directions in pursuing me and does not affect the houses. It seems the lava ball has the task of getting me. As the lava ball catches up, I wake up. It didn’t hurt anymore to breath in the hot air, only the lung, the air tube, and the nose was hurting.”

## Discussion

The present study indicates that fever affects dreaming; fever dreams are more bizarre – confirming the previous finding of our pilot study (Schredl et al., 2016b) in an independent sample – but also included more negative dream emotions, less dream characters and interactions, and more health-related topics and heat perceptions than the matched normal non-fever dreams. As fever dreams have not yet been studied systematically, it is reassuring that we were able to replicate the pilot findings with a new independent sample indicating that the present findings are substantial.

Before discussing the findings, several methodological issues will be addressed. First, fever dreams were elicited retrospectively, i.e., sometimes experienced quite some time ago. That might have biased the results as extraordinary dreams are more likely to be recalled after such long periods of time (Cipolli et al., 1992). However, the time interval between occurrence of the dream and its reporting was not related to emotional intensity or bizarreness. In addition, the dreams selected for comparison were also retrospectively recalled dreams. In order to test for possible recall effects using retrospective designs, it would be very interesting to use a prospective approach like Smith (2012b), i.e., hand out a dream diary and instruct the participants to complete this diary if they suffer from a febrile illness. However, one has to keep in mind that fever does not occur that often, so this study might be arduous. The retrospective nature of the study also does not allow any inferences regarding the sleep stage in which the dreams occurred. As fever can trigger episodes of sleepwalking (Avidan, 2017), one might speculate if, for example, the first dream example is a remembrance of a sleepwalking episode. Typically reports from NREM parasomnia episodes can include the bed chamber but are very brief (Arnulf, 2019), so the finding that fever dreams are generally comparable in length and even more bizarre than “normal” dreams indicates that those reports rarely reflect sleepwalking. However, content of sleepwalking episodes related to fever has never been studied systematically; the subjective experiences within these episodes might also be more bizarre compared to “normal” sleepwalking episodes. Due to the rare occurrence of fever episodes, ambulatory polysomnographic studies, i.e., recording the sleep stage prior to the reported dream, are very arduous. It would also be very interesting to study the effect of experimentally increasing body temperature by cytokines (cf. Reichenberg et al., 2001) on dream characteristics and content. Next, it should be noted that the sample consisted of high dream recallers; the mean dream recall frequency in the general population is about one morning per week with dream recall (Schredl, 2008) whereas in our study the mean dream recall frequency indicated dream recall several times a week. On the other hand, reporting a fever dream was not related to dream recall frequency but to the frequency of having fever. But one might argue that the reported percentages of experiencing fever dreams while being ill is an overestimation in this study due to overall heightened dream recall and, therefore, it would be necessary to carry out surveys in representative samples for obtaining data as to how often fever dreams occur.

The finding that fever dreams contain more intense negative emotions and less intense positive emotions supports the continuity hypothesis of dreaming as fever is also accompanied by more negative moods in waking (Reichenberg et al., 2001) and negatively toned dreams might reflect these negative waking emotions. This link between waking emotional tone and dream emotions has been shown in healthy persons (Schredl and Reinhard, 2009-2010). Also, Bódizs et al. (2008) found that poor health is related to more negatively toned dreams. To follow up this line of thinking, future studies could elicit mood during waking in persons with fever and test how strong waking emotions affect dreams while being ill. Similarly, it would be interesting to test whether the cognitive impairment in waking due to fever (Hall and Smith, 1996; Smith, 2012a) is directly related to dream bizarreness, i.e., are dreams of persons with more pronounced cognitive impairments more bizarre than dreams of persons with mild cognitive impairments during a febrile illness? The basic idea is that the “over-heated” brain is not functioning properly and, therefore, dreams are more bizarre. In schizophrenic patients, for example, the severity of psychotic symptoms during the day is directly related to dream bizarreness (Schredl and Engelhardt, 2001).

Also in line with the continuity hypothesis is the finding that fever dreams included more health-related topics. A previous study in patients with insomnia (Schredl et al., 1998b) showed that more health problems are associated with more health-related dreams. Interestingly, the frequency of health-related dreams is not only related to the frequency of the illnesses but also to worrying about health (Schredl et al., 2016a), i.e., future studies might also include this variable.

Interestingly, the findings of less dream characters and less physical and verbal Interactions also fit in the continuity hypothesis because one of the accompanying behavioral changes of fever is social withdrawal (Harden et al., 2015).

Lastly, fever dreams included more references to temperature perception (see the illustrative second dream example) than non-fever dreams. In a long dream series, explicit temperature perceptions were present in only in 0.63% of the dreams (Schredl, 2016). This increased number of temperature perceptions in fever dreams might reflect the waking-life experience of feeling hot, within the framework of the continuity hypothesis, but it is also plausible that the fever dreams might be affected by the internal sensation of feeling hot while sleeping. Research has shown that external stimuli like sounds, water spray, rocking of the bed, and mild pain stimuli are sometimes incorporated into dreams (Dement and Wolpert, 1958; Nielsen et al., 1993; Leslie and Ogilvie, 1996). Interestingly, somatosensory stimulation of the leg muscles was incorporated into dreams quite often and could result in bizarreness related to the body image (Nielsen, 1993); the dream examples might also reflect a very creative processing of the internal heat stimulus. However, studies on the effect on dreams of thermal stimulation, e.g., thermal stimuli applied to the skin, have not yet been performed. If heat stimuli are incorporated into dreams, the hypothesis that fever directly affects dreams via the increased body temperature would be supported.

To summarize, this study showed that fever dreams are quite common and differ significantly from non-fever dreams, i.e., fever dreams were more bizarre, more negatively toned and included more references to health and temperature perception. Future studies should follow up this line of research by conducting diary studies during naturally occurring febrile illnesses and sleep laboratory studies with experimentally induced fever. This research helps to understand subjective experiences while sleeping in an extreme condition.

## Data Availability Statement

The datasets generated for this study are available on request to the corresponding author.

## Ethics Statement

Ethical review and approval was not required for the study on human participants in accordance with the local legislation and institutional requirements. Written informed consent from the participants’ legal guardian/next of kin was not required to participate in this study in accordance with the national legislation and the institutional requirements.

## Author Contributions

MS and DE contributed to the conception and design of the study, manuscript revision, and read and approved the submitted version of the manuscript. DE organized the database. MS performed the statistical analysis and wrote the first draft of the manuscript.

## Conflict of Interest

The authors declare that the research was conducted in the absence of any commercial or financial relationships that could be construed as a potential conflict of interest.
